# Dietary Carotenoids Regulate Astaxanthin Content of Copepods and Modulate Their Susceptibility to UV Light and Copper Toxicity

**DOI:** 10.3390/md10050998

**Published:** 2012-04-27

**Authors:** Maria-José Caramujo, Carla C. C. R. de Carvalho, Soraya J. Silva, Kevin R. Carman

**Affiliations:** 1 Centre for Environmental Biology, Faculty of Sciences, University of Lisbon, Campo Grande C2, 1749-016 Lisbon, Portugal; 2 IBB-Institute for Biotechnology and Bioengineering, Centre for Biological and Chemical Engineering, Department of Bioengineering, Instituto Superior Técnico, Technical University of Lisbon, Av. Rovisco Pais, 1049-001 Lisbon, Portugal; Email: ccarvalho@ist.utl.pt; 3 Departamento de Oceanologia y Ciencias Costeras, Instituto Venezolano de Investigaciones Científicas, Km 11 Carretera Panamericana, Altos de Pipe, Estado Miranda, Venezuela; Email: ssilva@ivic.gob.ve; 4 Department of Biological Sciences, Louisiana State University, Baton Rouge, LA 70803, USA; Email: zocarm@lsu.edu

**Keywords:** astaxanthin, ROS, toxicity, copper, carotenoid pigment, copepod, HPLC

## Abstract

High irradiation and the presence of xenobiotics favor the formation of reactive oxygen species in marine environments. Organisms have developed antioxidant defenses, including the accumulation of carotenoids that must be obtained from the diet. Astaxanthin is the main carotenoid in marine crustaceans where, among other functions, it scavenges free radicals thus protecting cell compounds against oxidation. Four diets with different carotenoid composition were used to culture the meiobenthic copepod *Amphiascoides atopus* to assess how its astaxanthin content modulates the response to prooxidant stressors. *A. atopus* had the highest astaxanthin content when the carotenoid was supplied as astaxanthin esters (*i.e*., *Haematococcus* meal). Exposure to short wavelength UV light elicited a 77% to 92% decrease of the astaxanthin content of the copepod depending on the culture diet. The LC_50_ values of *A. atopus* exposed to copper were directly related to the initial astaxanthin content. The accumulation of carotenoids may ascribe competitive advantages to certain species in areas subjected to pollution events by attenuating the detrimental effects of metals on survival, and possibly development and fecundity. Conversely, the loss of certain dietary items rich in carotenoids may be responsible for the amplification of the effects of metal exposure in consumers.

## 1. Introduction

In the marine environment, reactive oxygen species (ROS) are relatively common and accumulate both in open and coastal waters [1]. ROS formation is favored by high irradiation, especially ultraviolet radiation (UVR) [1,2], oxygenic photoautotrophy [3,4] and by the presence of xenobiotics [5,6]. In aerobic organisms, which use oxygen (O_2_) for respiration or oxidation of nutrients, and in photosynthetic organisms which may transfer the excess energy from excited singlet chlorophyll on to the ever present O_2_, reactive by-products of oxygen are generated continuously in cells. Hydrogen peroxide, superoxide anion radical and the highly reactive hydroxyl radicals cause oxidative stress resulting in destructive effects on cellular macromolecules such as proteins, DNA, RNA and fatty acids [7,8,9]. In nucleic acids, both sugar and base moieties can be oxidized and the main oxidative damages include single and double strand breaks, modified bases and DNA-protein cross-links [9,10]. The target of ROS in cellular membranes is usually the polyunsaturated fatty acids, resulting in lipid peroxidation and consequently in a decrease in membrane fluidity, which changes membrane properties and may disrupt membrane-bound proteins [3,11]. ROS interaction with proteins may result in different damages such as oxidation of sulfhydryl groups, reduction of disulfides, modification of prosthetic groups and protein-protein cross-linking [12,13].

Through evolution, organisms have developed antioxidant defense mechanisms that prevent the formation of and capture ROS, and repair mechanisms for the oxidized compounds formed. The production of ROS may be prevented by affecting the transition metals in the Fenton reaction (e.g., iron and copper) with compounds such as metal chelators [14]. Specially adapted enzymes have also been used to prevent the deleterious effects of ROS, as well as a variety of non-enzymatic antioxidants that include ascorbic acid, reduced glutathione, flavonoids, aromatic amines and carotenoids [15,16].

Carotenoids are synthesized by photosynthetic organisms, bacteria and fungi whilst animals, in general, cannot produce them *de novo*. Animals obtain the necessary carotenoids either directly from the diet or modify the dietary carotenoid precursors through metabolic reactions to fit their requirements. The physiological functions of carotenoids in photo-autotrophs are related to the photosynthetic process. Carotenoids participate in the collection of light energy and its transfer to chlorophyll for photosynthesis [17,18]. In their role as photo-protectors, carotenoids both dissipate the excessive energy used in photosynthesis and inhibit the formation of ROS. In bacteria, carotenoids have also been linked to tolerance and adaptation to several stressful conditions including salinity, radioactive compounds, pH and temperature. In heterotrophic microorganisms, carotenoid production is not as essential as in photoautotrophic microbes which need protection from light [19], yet carotenoids are widely distributed in extremophiles. In these microorganisms, carotenoids are often accumulated in membranes where they contribute to the stabilization of the membrane under extreme conditions. The thermo(bis)zeaxanthins, for example, present a hydrophobic-hydrophilic-hydrophobic structure resulting in the positioning of zeaxanthin in the lipid bilayer, glucose at the surface of the membrane and branched fatty acids curled back into the lipid bilayer [20]. The importance of bacterial carotenoids in the environment has only recently been acknowledged. Antón and co-workers have demonstrated that bacteria may constitute from 5 to 25% of the total prokaryotic community in crystallizer ponds (salinity 30–37%) from multipond solar salterns [21]. Nevertheless, the major source of carotenoids to the marine food webs is considered to be from photosynthetic organisms. 

The importance of carotenoids in aquatic food webs was highlighted as shifts in nutrients were shown to alter the community structure of producers which in turn affected consumers such as fish and mammals. The reduced availability of thiamine (vitamin B1) and astaxanthin has been related to the M74 syndrome, which is a reproductive disorder of salmon (*Salmo salar* L.) in the Baltic Sea [22]. Thiamine and astaxanthin depletion result from changes in the inorganic nutrient dynamics which cause shifts in the phytoplankton community composition that, in turn, influence copepod population growth, and the availability of these compounds to higher trophic levels of the aquatic food web [23]. In marine ecosystems, astaxanthin is the main carotenoid produced by crustaceans from other algal carotenoids, especially β-carotene, which is considered to be its main precursor [24,25]. The oxidative pathways to convert β-carotene into astaxanthin may either involve the oxidation of β-carotene through echinenone and canthaxanthin or, through β-cryptoxanthin, zeaxanthin and adonirubin, as suggested to occur in species of the microalga *Haematococcus* [26]. Astaxanthin has a strong ability to quench singlet oxygen [27] and is a particularly strong scavenger of free radicals preventing the peroxidation of poly-unsaturated fatty acids (PUFA). The most important function of astaxanthin in copepods is that of an antioxidant for protecting lipids from peroxidation [28,29]. A second function as photoprotector has been acknowledged, since pigmentation of body tissues and eggs offers protection against photosynthetic active radiation (PAR) [30,31] and ultraviolet (UV) light [32,33]. Aquatic organisms are known to be susceptible to UVR which is reflected in suppressed reproduction and increased adult and juvenile mortality [34,35]. According to Ringelberg [36], a third function could be related to the use of astaxanthin esters as a source of metabolic energy, even if they contribute to only *ca.* 2% of the total lipid content of a copepod body [31]. It has been also suggested that, during the rapid combustion of lipid material in upwardly migrating copepods, astaxanthin could act as physiological “replacement” of the oxygen molecule as electron acceptor [37].

As the carotenoid content of aquatic organisms has implications for antioxidant protection, carotenoid content must also be of paramount importance to organisms subjected to sources of oxygen radicals like toxic contaminants. Metal toxicants, such as copper, can damage cells by promoting oxidative mechanisms, and as UVR, by generating ROS and free radicals (e.g., catalyzing the formation of highly reactive hydroxyl radicals [38]). However, the role of the carotenoid content of copepods in shaping the survival response to the exposure to prooxidant toxicant chemicals has been widely neglected in toxicity studies. As when organisms and cells are exposed to UVR, carotenoids may act as a physico-chemical barrier to copper deleterious effects by scavenging free radicals, thus protecting cell membranes against oxidation [37,39], and the genome from free radical-mediated damage [40]. It is therefore of ecological interest to explore how the availability of carotenoids in the diet [41] shape the response of aquatic consumers to UVR and heavy metal exposure, and how these stressors interact. 

In this study, we explore how the carotenoid content of the meiobenthic copepod *Amphiascoides atopus* modulates the survival to UVR and copper exposure, and how these prooxidant stressors interact. Additionally we test whether the dietary source of astaxanthin, directly obtained or synthesized from precursors in the diet determines the efficiency of astaxanthin protection from the prooxidant agents.

## 2. Results and Discussion

### 2.1. Effect of Diet on the Astaxanthin Content of Copepods

**Figure 1:**
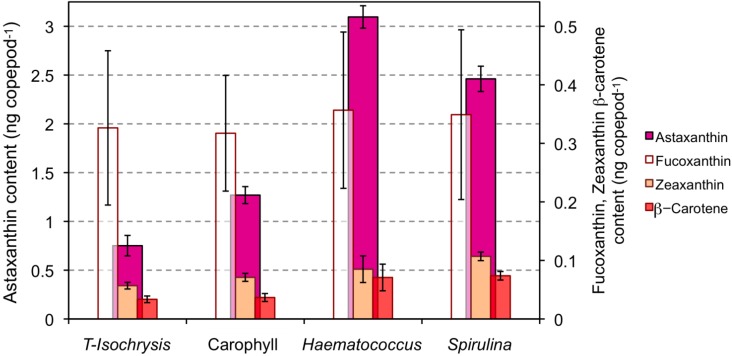
Carotenoid content (ng individual^−1^) of copepods cultured under photosynthetic active radiation (PAR) light and on the four types of diet: T-*Isochrysis* paste plus enriched torula yeast (T-*Isochrysis*), T-*Isochrysis* paste plus enriched torula yeast plus artificial astaxanthin (Carophyll), T-*Isochrysis* paste plus *Haematococcus* meal (*Haematococcus*) and T-*Isochrysis* paste plus *Spirulina* meal (*Spirulina*).

The astaxanthin content of copepods grown under PAR light significantly increased when the diet of T-*Isochrysis* plus enriched torula yeast was supplemented with astaxanthin or astaxanthin precursors (Figure 1; F_3,16_ = 371.6, *p* < 0.001). The highest astaxanthin content was observed for the copepods feeding on the diet supplemented with *Haematococcus* (3.10 ng copepod^−1^ or *ca.* 619 μg g^−1^ dry weight), and the lowest astaxanthin content was observed for copepods feeding on the diet supplemented with free astaxanthin, Carophyll^®^ Pink (1.27 ng copepod^−1^; Tukey’s B *p* > 0.05; Figure 1). Copepods feeding on the diet supplemented with *Spirulina* meal had the highest zeaxanthin content (F_3,16_ = 9.6, *p* = 0.002). The β-carotene content of copepods feeding on the diet supplemented with either *Haematococcus* or *Spirulina* meals was significantly higher than the content of copepods feeding on either of the other two diets (F_3,16_ = 10.6, *p* = 0.001; Tukey’s B *p* > 0.05). Fucoxanthin content of copepods was similar with all diets.

*A. atopus* had a carotenoid composition similar to other copepods [23,41,42,43,44]. Copepods increased their carotenoid content up to five-fold depending on the availability of carotenoids in the diet which shows that copepods are able to profit from carotenoid enriched diets [23,44]. The level of increase is on the same scale to that observed for *Nitocra lacustris*, a marine copepod (5.5 μg dry weight) that increased its astaxathin content from 1.0 ng copepod^−1^ when feeding on the prasinophyte *Tetraselmis suecica* to 3.6 ng astaxanthin copepod^−1^ when feeding a formulated feed containing lycopene, α-carotene, β-carotene, lutein, phytoene and phytofluene [44]. 

*A. atopus* incorporated more astaxanthin supplied mainly in the esterified form (*Haematococcus* meal) than when supplied in free form Carophyll^®^ Pink. Astaxanthin is deposited mainly as esters and not as free astaxanthin in many crustaceans, particularly during ontogenetic development [37,43]. Copepods may either digest, assimilate or incorporate preformed astaxanthin esters more efficiently than free astaxanthin [45,46], or have a preference for the stereoisomer 3*S*,3′*S* that is more abundant in *Haematococcus* meal (>99% 3*S*,3′*S* [47]) than in Carophyll^®^ Pink (18.75% 3*S*,3′*S* [46]). The importance of variations in carotenoid assimilation dependent on food concentration to shape the degree of astaxanthin accumulation cannot be dismissed [48]. Nevertheless, it is likely that the observed differences in copepod astaxanthin content reflect the importance of food quality, in terms of stereoisomers or chemical forms of carotenoids, for its digestibility and incorporation by copepods. The absence of astaxanthin in *Spirulina* meal and the large content of astaxanthin in copepods feeding on *Spirulina* points to bioconversion from precursor carotenoids by the copepods, possibly from β-carotene which is present in large amount in the diet [49]. It has been demonstrated that some crustaceans are able to bioconvert zeaxanthin into astaxanthin via β-doradexanthin [50], yet this carotenoid was not identified in our analysis. Therefore, this pathway is not, apparently, used by *A. atopus* to obtain astaxanthin from the abundant zeaxanthin in the *Spirulina* meal.

### 2.2. Effect of Light Type Exposure on the Astaxanthin Content of Copepods

**Figure 2:**
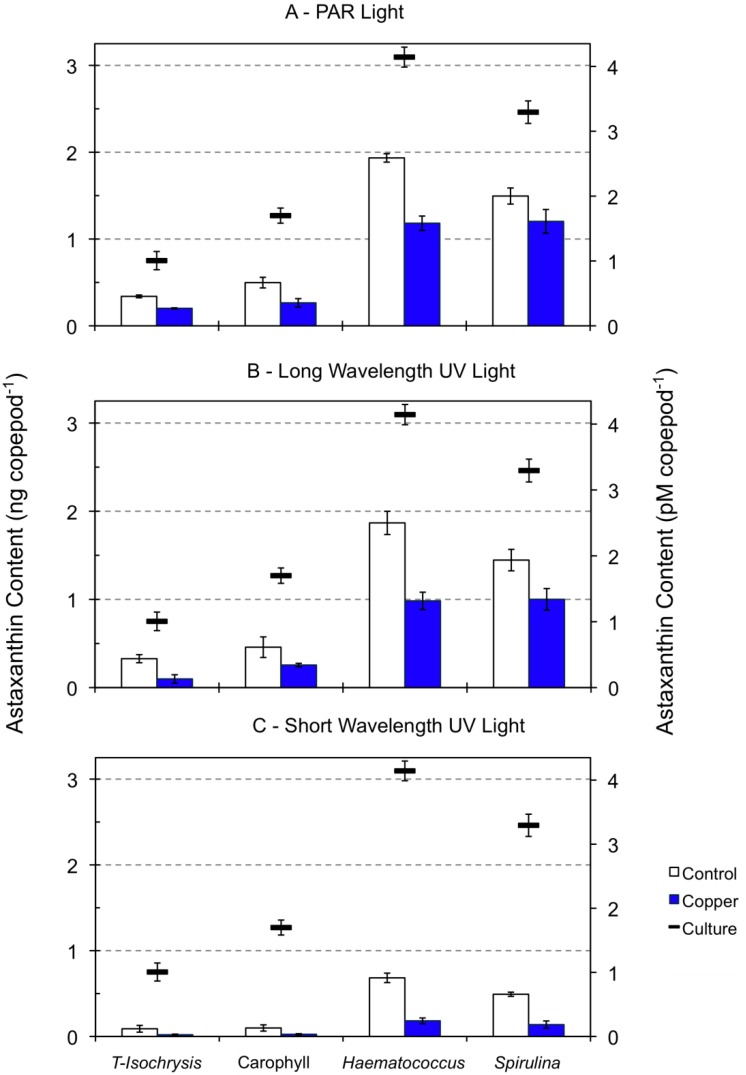
Astaxanthin content of copepods (ng and pM copepod^−1^) fed on four types of diet (see Figure 1 legend). Dashes indicate the astaxanthin content of copepods in cultures under PAR light while columns indicate the astaxanthin content of 96 h starved copepods exposed to PAR light (panel A), long wavelength UV light (panel B) and short wavelength UV light (panel C), in the absence and presence of copper at a concentration of 2 to 5 μM.

Zeaxanthin and β-carotene were reduced by *ca*. 50%, and fucoxanthin was detected below the quantification limit in 96-h starved copepods during the acute toxicity tests under PAR and long wavelength UV light relative to cultured copepods (data not shown). These pigments were detected below the quantification level in starving copepods exposed to short wavelength UV light for 96-h. The decrease in the astaxanthin content of copepods starved for 96 h and exposed to PAR and long wavelength UV light ranged from around 40% (minimum of 37% for the diet supplemented with *Haematococcus* meal) to 63% (diet supplemented with Carophyll^®^ Pink; Figure 2). When exposed to short wavelength UV light, starving copepods exhibited a severe astaxanthin decrease, ranging from 77% (diet supplemented with *Haematococcus* meal) to 92% (diet supplemented with Carophyll^®^ Pink). The astaxanthin content of 96-h starved copepods was significantly dependent on both the diet type used in the cultures (F_3,38_ = 260.1, *p* < 0.001) and the type of light exposure (F_2,38_ = 150.0, *p* < 0.001). A significant interaction between diet and light was also found (F_6,38_ = 16.7, *p* < 0.001). 

The reduction of β-carotene and zeaxanthin by *ca*. 50% in the 96-h starved copepods under PAR and long wavelength UV light relative to the start of the experiments may be either the result of its direct metabolic use, or its bioconversion to compensate for astaxanthin loss due to starvation. The presence of β-carotene in trace amounts and the great reduction of astaxanthin in copepods exposed to short UV light suggest the destruction of pigments during their utilization as protectants against photooxidation, as described for tide pool dwelling harpacticoid copepods in high radiation environments [41]. Oxidative degradation of carotenoids occurs in various biological systems, and the rate of oxidation decreases from β-carotene or zeaxanthin to astaxanthin [51].

Carotenoids possess a polyene chain, which is a long conjugated double bond system forming the backbone of the molecule. The polyene chain may be terminated by cyclic end groups that contain oxygen-bearing substitutes (e.g., astaxanthin), and is responsible for the antioxidant activities of the carotenoids, both by quenching singlet oxygen [52], and scavenging radicals to terminate chain reactions [53]. This mechanism of UVR protection offered by carotenoids agrees with that proposed by Sommaruga [54], who suggested that the mechanism by which carotenoids function is likely not direct photoprotection (via reflectance or absorbance of UVR) but rather indirect photoprotection by scavenging ROS formed by UVR. Ringelberg [36] noted that carotenoid compounds are conspicuously found within copepod fat globules, carapace epidermis, ovaries, and eggs, which are areas critical to survival, and highly prone to the damaging effects of UVR induced ROS. The proposed main function of astaxanthin in crustaceans, especially in the form of astaxanthin esters, is to generally improve the antioxidant protection of storage lipids, also in situations where photoprotection is not required [55]. 

The protection offered by carotenoids may be especially important against lipid peroxidation in cellular membranes, and polar carotenoids like astaxanthin are known to play a role in preserving membrane structure and reducing lipid hydroperoxide levels [56]. In membranes, polar carotenoids appear to span the membrane with their polar end groups extending toward the polar regions of the membrane bilayer, spanning the membranes in a parallel fashion, increasing the order parameter of the membrane bilayer [57]. This, in turn, restricts the permeation of the membrane to polar molecules and ions [58,59]. Astaxanthin has a molecular length similar to membrane bilayers, and also possesses a ketone group at the C4 and C4′ positions of the terminal rings, which may act to further stabilize astaxanthin's membrane interactions with respect to the polar terminal groups [57]. By spanning the entire width of the membrane as proposed by Woodall *et al*. [39], astaxanthin would enhance antioxidant activity by providing protection throughout the entire depth of the membrane, interfering with the propagation of free radicals in the hydrophobic core, and quenching radicals generated at the surface of membranes [57].

### 2.3. Effect of Copper Exposure on the Astaxanthin Content of Copepods and Their Survival

Fucoxanthin, zeaxanthin and β-carotene were detected below the quantification level in copepods exposed to copper at a concentration of 1 to 5 μM under all light types (data not shown). Under both PAR and long UV radiation, the effect of the diet was more significant on copepod astaxanthin content than the copper effect (Table 1, Figure 2). However, under short wavelength UV light, the opposite was observed (F_3,24_ = 246.1, *p* < 0.001 for diet effects and F_1,24_ = 432.7, *p* < 0.001 for copper effects; Table 1). The interaction between dietary and copper effects on the astaxanthin content of copepods was significant under all light regimes, and the strongest interaction was observed for copepods under short UV light (Table 1). The decrease in the astaxanthin content of copepods exposed to copper relative to control copepods under PAR light was lower for copepods fed on either diet supplemented with *Haematococcus* meal (39%) or *Spirulina* meal (20%) than for copepods feeding on the unsupplemented diet (41%) or the diet supplemented with Carophyll^®^ Pink (47%, Figure 2). Nevertheless, copepods feeding on the diets supplemented with either *Haematococcus* or *Spirulina* had a higher absolute astaxanthin decrease, which suggests an increased use of astaxanthin when it is more available in the body tissues. The same pattern was observed for copepods tested under long UV light. The decrease in astaxanthin content of copepods exposed to copper under short UV light was similar with all diets, ranging from 78% for the unsupplemented diet to 72% for the diet supplemented with *Spirulina* meal.

**Table 1:** F values from ANOVAs applied to astaxanthin content of copepods grown under four types of diet (see Figure 1 legend) and exposed to 96-h acute toxicity tests using copper at 1 to 5 μM (63.55 to 317.73 μg L^−1^). All *p* < 0.001, except for F * where *p* = 0.002.

Tested Effect	PAR (*n* = 24)	Long UV radiation (*n* = 26)	Short UV radiation (*n* = 24)
Diet (df = 3)	179.3	162.6	246.1
Copper (df = 1)	51.8	83.0	432.7
Diet × Copper (df = 3)	7.8 *	11.1	79.5

Copper (Cu) is an essential micronutrient for all living organisms, being involved in cellular respiration, free radical defense and cellular iron metabolism. Yet, at elevated levels, Cu is toxic to organisms and there is evidence that *in vivo* formation of reactive oxygen species is a mechanism of copper toxicity [60]. The astaxanthin content of copepods starved for 96 h under PAR or long UV light was more dependent on the diet offered than on the exposure to low copper concentrations (1–5 μmol). The higher dependence of the astaxanthin content on copper exposure than on the original diet under short UV light, points to the role of copper as a synergistic or additive factor to the natural environmental stressor UV light. In turn, short UV light interacts with copper to increase its destructive potential, thus increasing the influence of copper on copepod astaxanthin content to a higher level than that of the diet. The strong decrease of the astaxanthin content of copepods during both copper and short UV light exposure may have resulted from oxidative degradation that caused the disruption and breakdown of the polyene chromatophore [61]. This bleaching of pigments in crustaceans is often observed when animals are exposed to trace metals.

**Figure 3:**
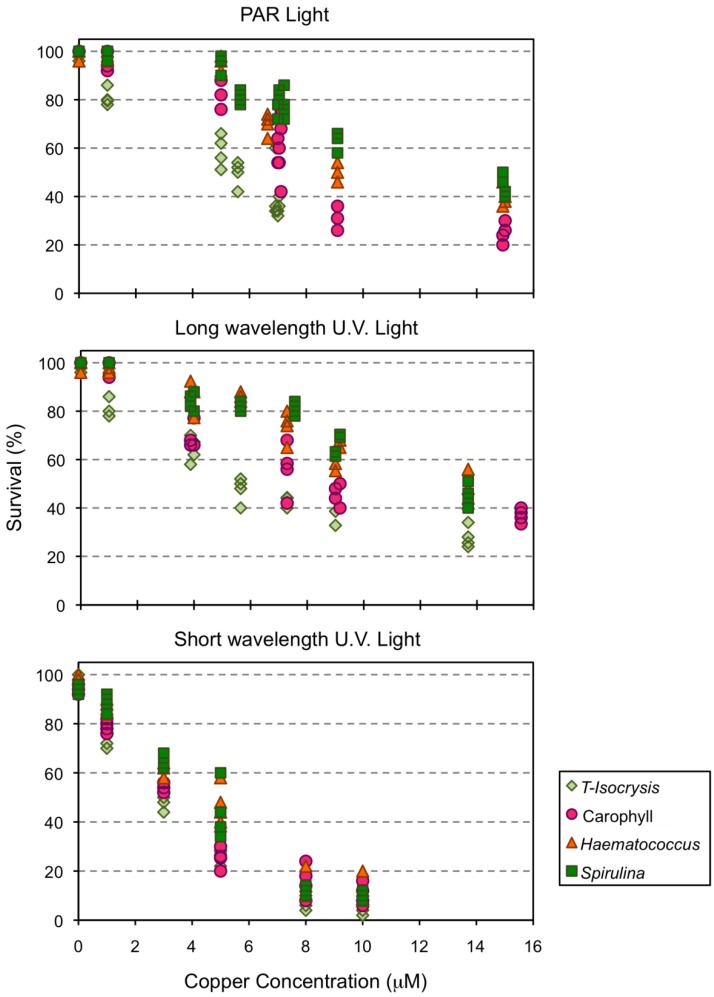
Survival of the copepod *Amphiascoides atopus* after 96 h exposure to different concentrations of copper (μM).

Mortality of *Amphiascoides atopus* ranged from 0–4% in control artificial sea water (ASW) under both PAR and long UV light, and from 0–8% under short wave UV light. Clear dose-response relationships were observed for survival after 96 h exposure, although the intensity of response under both PAR and long wavelength UV light was modulated by the type of diet used to culture the copepods (Figure 3). The culture diet had a smaller effect on the response of copepod survival to the exposure of copper under short wave UV light than under the other light types (Figure 3).

**Figure 4:**
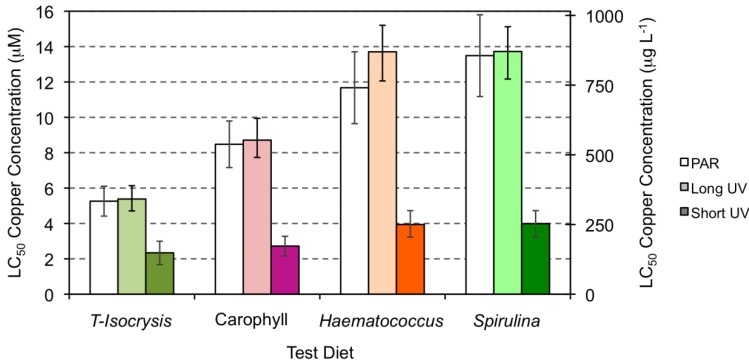
Estimates of the LC_50_ for copper exposure of copepods tested under PAR light (white), long UV (light color) and short UV light (strong color), and fed on the four test diets (see Figure 1 legend). Error bars express the lower and upper boundary of the 50% mortality response.

Estimates of the LC_50_ for copper were higher when the copper exposure occurred under PAR or long UV light (5.26–13.72 μM or 334.24-872.02 μg L^−1^; Figure 4) than when the tests were conducted under short wave UV light (2.34–3.98 μM or 148.71–253.50 μg L^−1^; Figure 4 and Table 1). The LC_50_ values and the corresponding upper and lower limits were higher when the copepods had been cultured under diets supplement with either *Haematococcus* meal or *Spirulina* meal, irrespective of the type of light exposure. Under both PAR and long wavelength UV light, the culture diet supplemented with Carophyll enabled an LC_50_ of copper that was intermediate between the unsupplemented diet (Figure 4, T-*Isochrysis*) and diets supplement with either *Haematococcus* meal or *Spirulina* meal.

The protection offered by astaxanthin was relevant, in terms of survival, when *A. atopus* was exposed to the ROS generating copper toxicant. *A. atopus* mortality varied directly with Cu concentration, and the LC_50_ 96 h for copper increased with the increase of copepod carotenoid content. The higher mortalities were observed when the copepods were exposed both to Cu and short UV light, and only the diets supplemented with *Spirulina* or *Haematococcus* were able to ascribe some degree of protection to the copepods. Bell *et al*. [62], using Carophyll pink as a source of astaxanthin to Atlantic salmon, observed that the carotenoid had antioxidant functions, offering protection against lipid peroxidation. The absence of protection against both copper and short UV exposure in the present experiment may simply be the result of inefficient assimilation and incorporation of artificial astaxanthin by copepods. 

*A. atopus* is less sensitive to Cu than *Tigriopus brevicornis* and *Tisbe battagliae* and more sensitive to Cu than *Tigriopus californicus*, *T. japonicus* and *Tisbe holothuriae* (see Table 2). *A. atopus* is also more sensitive to cadmium (LC_50_ 96 h, 4.13 ± 0.75 μMol Cd; [63]) than to copper (LC_50_ 96 h, 4.42–6.23 μMol Cu), although the lower limit of the LC_50_ of Cu overlaps with that of Cd. The lower sensitivity to copper relative to cadmium is the reverse to that observed for *Tigriopus japonicus* [64] and for *Tisbe holothuriae* [65]. Nevertheless, the latter harpacticoids were more resistant to the toxicity of both metals than *A. atopus* (see Table 2). The resistance to metals differs widely among harpacticoid species, different life stages and different geographically isolated populations, and some variations may also have been introduced by the quality of food used in some studies. The standardization of culture procedures and food for copepods used in toxicological tests [66,67] is an important step to obtain reliable results that allow comparisons among species and life stages. 

The diet-dependent UV light resistance in high shore harpacticoid copepod *Tigriopus brevicornis* has been noted [41] and it is likely that the presence of astaxanthin gives an adaptive advantage to populations of astaxanthin rich species in copper-enriched environments where most intertidal seaweed and macroinvertebrate populations are eliminated [68]. Conversely, it has been observed that some harpacticoid species that are not acutely sensitive to most common pollutants, greatly increase their sensitivity after chronic exposure, especially in terms of population parameters [69]. Such observations, and the results presented here, point to the importance of copepod health and nutrition status during and after being subjected to toxicants.

**Table 2:** Comparative data of copper acute toxicity LC_50_ 96 h (μM and μg L^−1^) in marine harpacticoid copepods with reference to food offered prior to the toxicity experiments (starving during toxicant exposure). Lower and upper boundaries are given for LC_50_ 96 h values calculated in the present study; LC_50_ 96 h for cadmium are given in parentheses.

Species	Stage	Food	Cu (μM)	Cu (μg L^−1^)	Reference
Amphiascoides atopus (under PAR light)	Adult	A = T-Isocrysis + enriched Torula yeast	5.26(4.42–6.23)	334.24(280.6–396.1)	Present study
		A + Carophyll	8.48(7.16–10.35)	538.67(455.0–657.8)	
		A + Haematococcus	11.67(9.64–15.66)	741.83(612.7–995.3)	
		A + Spirulina	13.49(11.17–15.90)	857.19(710.3–1204.6)	
A. atopus	Adult	T-Isocrysis	(Cd = 4.88 ± 0.75)	(Cd = 549 ± 84)	[63]
Nitocra spinipes	Adult	Unknown	28.33	1800	[70]
Tigriopus brevicornis	Adult	Field collected, unfed	2.36	150	[71]
Tigriopus californicus		Fish food (Wardley’s Basic Food Flakes)	11.99	762	[72]
Tigriopus japonicus	Adult	Tetraselmis suecica	61.37(Cd = 24.18)	3900(Cd = 25,200)	[64]
Tigriopus japonicus	C5–C6	Field collected, unfed	16.11	1024	[73]
Tigriopus japonicus	Adults	Enteromorpha spp. plus phytoplankton concentrate (Kent Marine Phytoplex, US)	12.79	813	[74]
Tisbe battagliai ^1^	Adult	Artificial (Marine Invertebrate Diet™, Hawaiian Marine Imports Inc.)	2.47	157	[75]
Tisbe holothuriae ^2^	Females with ovigerous bands	Unknown	7.04(Cd = 8.15)	447.3(Cd = 916.6)	[65]
	Females with ovisacs	Unknown	6.74(Cd = 7.76)	428.2(Cd = 872.7)	

^1^ LC_50_ 72 h; ^2^ LC_50_ 48 h.

## 3. Experimental Section

### 3.1. Cultures

Cultures of *Amphiascoides atopus* Lotufo and Fleeger were obtained from laboratory cultures kindly supplied by Dr. John W. Fleeger. The cultures were established in 1992 and have been continuously maintained since that date in sediment free, 1 L Erlenmeyer flasks at room temperature (23 °C [76]). Adult female copepods reach up to 5 μg dry weight [77]. Since the members of this genus typically live in beaches with coarse sediment and are not associated with muddy environments, all experiments were conducted free of sediment. For the present experiments, cultures were started with more than 50 males and 50 females grown in artificial sea water at 30‰ (ASW, Instant Ocean^®^ Sea Salt, USA), and fed every three days with T-*Isochrysis* paste (100 μL L^−1^; Brine Shrimp Direct, Odgen UT, USA) plus enriched torula yeast diet (10 mg L^−1^; Microfeast Plus^®^ L-10, Provesta Corp., OK, USA). T-*Isochrysis* paste is rich in poly-unsaturated fatty acids, especially docosahexaenoic acid (DHA [78]), and enriched torula yeast offers a diet rich in fatty acids, protein, minerals and B-vitamins (manufacturer specifications). T-*Isochrysis* paste has been individually used to successfully culture *A. atopus* in the laboratory and during toxicological studies [63], and enriched torula yeast has been used to mass culture the harpacticoid copepod *Tisbe* sp. [79]. After a week, the adults were removed by sieving the cultures through a 125-µm aperture screen and the nauplii were allowed to grow under photosynthetic active radiation (PAR) with a photoperiod of 12 h dark:12 h light and were fed one of the following test diets: (i) T-*Isochysis* = T-*Isochrysis* paste (100 μL L^−1^) plus enriched torula yeast (10 mg L^−1^); (ii) Carophyll = T-*Isochrysis* paste (100 μL L^−1^) plus enriched torula yeast (10 mg L^−1^) plus free astaxanthin (0.5 mg L^−1^ Carophyll^®^ Pink, Hoffmann-La Roche, Basel, Swizerland); (iii) *Haematococcus* = T-*Isochrysis* paste (100 μL L^−1^) plus *Haematococcus* meal (5 mg L^−1^ NatuRose™, Cyanotech Corp., HI, USA); (iv) *Spirulina* = T-*Isochrysis* paste (100 μL L^−1^) plus *Spirulina* meal (5 mg L^−1^ Spirulina Pacifica^®^, Cyanotech Corp., HI, USA). The food items used as a supplement of T-*Isochrysis* paste were chosen for their ability to offer a diet rich in astaxanthin (3,3′-dihydroxy-β,β′-carotene-4,4′-dione) or astaxanthin precursors. Carophyll^®^ Pink is nutritionally poor since the microbeads contain astaxanthin embedded in a matrix of gelatine and carbohydrate, enveloped by maize starch, according to manufacturer specifications. Both *Haematococcus* meal and *Spirulina* meal have additional nutritive value because of their amino acid and vitamin content [45,49]. The carotenoid pigments present in the supplements were extracted and analyzed by high performance liquid chromatography (HPLC) as described below for copepod total extracts. T-*Isochrysis* paste is a source of fucoxanthin and β-carotene, enriched torula yeast contains a small amount of astaxanthin, Carophyll^®^ Pink is a source of highly concentrated free astaxanthin (*ca*. 8% weight) containing approximately 75% of the natural all-E isomer [46], *Haematococcus* meal is mainly a source of astaxanthin in the mono (70%) and di-esterified (10%) form [45], and *Spirulina* meal is mainly a source of zeaxanthin and β-carotene (Table 3). Before adding the supplement diet to the food suspension, Carophyll^®^ Pink, *Haematococcus* and *Spirulina* meals were dispersed in ASW using a sonifier (Branson Sonifier 450, 3 mm diameter probe, output set on 4, duty cycle on 60%; Branson Ultrasonics, Danbury CT, USA). Previous pilot culture experiments have shown that the food concentrations offered to the copepods were in excess of the amount ingested for a period of 3 days, and all cultures were continuously reproducing.

**Table 3:** Carotenoid content and composition of food used to culture *A. atopus*. Total carotenoid content is given as average weight ± 1 S.D. per food dry weight (DW).

Food	Carotenoid content(µg mg^−1^ DW)	Carotenoid Composition (weight%)	Astaxanthin Isomers
T-Isochrysis paste(Brine Shrimp Direct)	3.18 ± 0.11	58%–Fucoxanthin 24%–Diadinoxanthin 3%–Diatoxanthin 15%–β-carotene	–
Enriched Torula yeast(Microfeast Plus^®^ L-10)	0.54 ± 0.07	87%–Astaxanthin 3%–Echinenone 2%–β-carotene	Unknown
Carophyll^®^ Pink	81.12 ± 1.09	100%–Astaxanthin	18.75%–(R,R′) 18.75%–(S,S′) 37.50%–(R,S) 25%–Z-isomers [46]
Haematococcus meal(NatuRose™)	17.86 ± 0.96	84%–Astaxanthin 2%–Canthaxanthin 7%–Lutein 2%-β-carotene	>99%-(S,S′) [47]
Spirulina meal	3.36 ± 0.10	23%–Zeaxanthin 10%–Echinenone 5%–β-cryptoxanthin 54%–β-carotene	-

Adults were harvested from the experimental cultures after 2–3 weeks by sieving the culture medium through a 125-µm aperture screen and the retained newly molted females (<48 h) were sorted under a stereo-dissection microscope. By selecting cohorts of a single development stage (*i.e*., newly molted females), we have reduced variations in copepod carotenoid content related to copepod ontogenic stage [37]. Females were placed in filtered (2 μm) ASW for 4 h to empty their gut, and ASW was changed twice at 1.5 h intervals. Groups of 50 females grown on all types of diets were placed in 100 mL plastic vials for 96-h acute toxicity tests (see below). Groups of 200–300 females from all cultures were placed over glass GF/C Whatman filters, subjected to a gentle vacuum suction to remove culture medium and rinsed with ASW filtered through a 0.2 mm pore size filter. The filters with the females were placed in extraction vials for carotenoid extraction and analysis (see below). 

### 3.2. Pigment Extraction and Analysis

The filters containing the copepods were placed in extraction vials on ice and pigments were immediately extracted in 1 mL 100% HPLC grade acetone (Fisher Scientific) using a sonifier cell disruptor for 30 s (Branson Sonifier 450, 3 mm diameter probe, output set on 4, duty cycle on 80%; Branson Ultrasonics, Danbury CT, USA). The extracts were bubbled with nitrogen gas to remove oxygen, incubated overnight in the dark at 4 °C and then filtered using syringe filters (Sun International; diameter 13 mm; 0.2 mm pore size) to remove debris. The filtered extracts were dried under nitrogen gas and the pigments were re-suspended in 200 mL acetone and stored in the dark at −80 °C before enzymatic hydrolysis of astaxanthin esters or direct pigment analysis, which took place within 24 h.

Half of the extracts from copepod cultures under the four types of diet and all pigment extracts of copepods subjected to the 96-h acute toxicity tests were hydrolysed by an enzymatic procedure to yield free astaxanthin. The enzyme used was cholesterol esterase from *Pseudomonas fluorescens* (Sigma-Aldrich, MO, USA) and the procedure followed Jacobs *et al*. [80].

The pigments in whole and hydrolysed extracts were analyzed using a Hewlett Packard 1100 High Performance Liquid Chromatograph (HPLC) with a 100 µL loop autosampler and a quaternary solvent delivery system coupled to a diode array spectrophotometer. The diode array detector was set at 436 nm for detection of all carotenoid pigments, and at 478 nm for the detection and quantification of astaxanthin and astaxanthin esters. Separation of pigments was performed by reversed-phase liquid chromatography using a Adsorbosphere™ C18 column (5 µm particle size silica, 250 mm × 4.6 mm i.d., Alltech Associates Inc., USA) coupled to a guard column. Analytical separation of pigments was achieved by gradient delivery of three mobile phases adapted from Kraay *et al*. [81]. Peak analysis was performed using the Hewlett Packard ChemStation software. Pigments were identified by comparing their retention time and absorption spectra with those of authentic standards (Sigma-Aldrich Inc., USA), and pigment concentration was calculated relating its peak area in the recorded chromatogram with the corresponding area of calibrated pigment standards. Pigment concentration was expressed as micromoles per copepod (μM individual^−1^).

### 3.3. 96-h Acute Toxicity Tests

Copper solutions were prepared with Cu(II) chloride (reagent grade) and 30 ± 0.5‰ ASW previously filtered through a 0.2 µm pore size filter at 7 nominal concentrations: 0 (control), 1, 3, 5, 10, 15 and 20 μM (0, 63.55, 190.64, 317.73, 635.46, 953.19, 1270.92 μg L^−1^). Water samples were collected after one hour of preparation and at end of each experiment in order to analyze the actual toxicant concentrations in the water. Samples were collected without disturbing the precipitate that could be present at the end of the experiment. The samples were analyzed on an Inductively Coupled Plasma–Optical Emission Spectrometer (ICP-OES, Perkin Elmer 2000 DV, Perkin Elmer Inc., USA). From the actual toxicant concentrations in the water at the start and the end of each survival test, average exposure concentrations were calculated assuming an exponential decrease of the toxicant concentration over time. All vials used to conduct bioassays were acid cleaned prior to use. 

The copepods were subjected to three light treatments: PAR light with a photoperiod of 12 h dark: 12 h light (treatment 1); PAR light regime with the addition of 4 h of ultraviolet (UV) radiation with a wavelength of 365 nm (long wavelength or UV-A) at the middle of the PAR light period (treatment 2); PAR light regime with the addition of 4 h of ultraviolet (UV) radiation with a wavelength of 254 nm (short wavelength) at the middle of the PAR light period.

The 50 test female copepods were placed in each test vial using a glass pipette (four replicates per concentration and light treatment). After 96-h exposure, copepods were removed with a pipette to a shallow glass dish and the 50 test copepods were enumerated as live or dead. Copepods immobilized were considered alive if they displayed body motion when touched with a probe or exhibited clear motion of the digestive tract. 

A 50% lethal concentration (LC_50_) and 95% confidence interval for the contaminant were calculated from dose response mortality data by a non-linear curve-fitting procedure using Probit analysis (SPSS Statistical Package, SPSS Inc., USA). All LC_50_ estimates from sediment toxicity tests were based on measured contaminant concentrations.

After the 96-h acute toxicity tests, three or four groups of 80–100 live copepods pooled from the 5 μM nominal copper concentrations were individually picked from the counting vials with a glass pipette, placed on GF/C Whatman filters, subjected to a gentle vacuum suction to remove the medium and rinsed with ASW filtered through a 0.2 mm pore size filter. Pigment extraction followed the same procedure applied to copepods from cultures.

### 3.4. Calculations and Statistics

ANOVA were applied to astaxanthin molar data of both algae and copepods (SPSS Statistical Package, SPSS Inc., USA). Data were square root transformed to meet the requirement of homoscedasticity when group variances were proportional to the means. Unplanned comparisons were made using Tukey’s B test (α = 0.05) or Hochberg’s GT2 tests (in the case of unequal sample size) to identify homogeneous sub-sets. 

Survival was expressed as percentage of the corresponding controls and plotted against the actual toxicant concentration in the seawater. From the obtained dose response plots, LC_50_ values and their corresponding 95% confidence limits were calculated by a non-linear curve-fitting procedure using Probit analysis (SPSS Statistical Package, SPSS Inc., USA).

## 4. Conclusions

Copepods were able to increase their carotenoid content depending on the availability of carotenoids in the diet, thus profiting from carotenoid enriched diets. It is likely that copepods have preferences for certain dietary forms of astaxanthin or astaxanthin precursors that favor its digestion, assimilation and bioconversion or direct incorporation into the copepods tissues. Future research on copepod digestion and assimilation abilities and preferences (e.g., enzymatic selectivity) for certain stereoisomers or chemical forms of carotenoids should clarify this issue.

The protection offered by astaxanthin was relevant, in terms of survival, when *A. atopus* was exposed to the ROS generating copper toxicant. The direct dependence of copepod carotenoid content on dietary availability, and its repercussions for copepod survival, point to the importance of copepod health and nutrition status during exposure to toxicants.

The strong decrease of the astaxanthin content of copepods during both copper and short UV light exposure points to oxidative degradation of the carotenoid, which may be confirmed by the future identification of astaxanthin degradation products in copepods exposed to metal toxicity.

Harpacticoids are an important link between the phytoplankton or phytobenthos and higher trophic levels, and play an important role in the marine meiobenthic food web, especially as food for juvenile fish [82]. It would be interesting to clarify the importance of the transfer of carotenoids from harpacticoids to fish larvae in coastal areas, especially when contaminants are present. 
